# The Macroscopic Stress–Macroscopic Strain Relationship of the Hierarchical Honeycomb Nanoporous Materials by the Spherical Nanoindentation Simulation

**DOI:** 10.3390/nano15070544

**Published:** 2025-04-03

**Authors:** Fue Han, Hongwei Zhang, Jingnan Zhang

**Affiliations:** School of Urban Planning and Municipal Engineering, Xi’an Polytechnic University, Xi’an 710048, China; zhanghongwei@xpu.edu.cn (H.Z.); 20190406@xpu.edu.cn (J.Z.)

**Keywords:** macroscopic stress–macroscopic strain relationship, hierarchical honeycomb nanoporous material, spherical nanoindentation, uniaxial compression

## Abstract

The spherical nanoindentation macroscopic stress–macroscopic strain relationship of hierarchical honeycomb nanoporous material is defined by combining the spherical nanoindentation simulation and the uniaxial compression simulation. At the same time, the macroscopic elastic modulus and the macroscopic yielding stress of the hierarchical material are obtained from the curves through different methods. The results show that the macroscopic stress–macroscopic strain curve of the hierarchical nanoporous materials nanoindented to a depth of 30 nm is basically consistent with the curve of the hierarchical nanoporous materials when uniaxially compressed down to 25 nm. Through the nanoindentation and uniaxial compression, the macroscopic elastic moduli and the macroscopic yielding stresses are also close to the scale formula.

## 1. Introduction

Due to differences in pore size, hierarchical honeycomb nanoporous materials have better practical applications than single-porous nanoporous materials in all respects. However, there are few studies on the stress–strain relationships of hierarchical honeycomb nanoporous materials through the spherical nanoindentation; thus, the functions of hierarchical nanoporous materials are limited to a certain extent. 

Usually, the stress–strain (σ-ε) relationship of a material is obtained via uniaxial tensile or uniaxial compression tests. The elastoplastic mechanical properties of the material can also be measured from the relationship curves. However, for small-sized components that cannot be prepared for standard tensile or compression specimens, the material properties of the small-sized components mentioned above need to be obtained by other means. So far, it has been the intent of many scholars to obtain the corresponding stress–strain curves of small-sized specimens through spherical nanoindentation technology which is consistent with the relevant properties obtained by standard tensile tests or compression tests [1]; moreover, the mechanical properties of small-sized materials are finally obtained from the σ-ε curves. Pathak et al. [2,3,4,5] developed a method for extracting the spherical indentation σ-ε relationship from the corresponding indentation force–depth curve, and the indentation elastic modulus and the indentation yielding stress were obtained from the indentation σ-ε relationship. Weaver et al. [6] compared the Al-6061 spherical indentation σ-ε relationship with the results of the uniaxial tensile test, and found that the scale factor between the uniaxial tensile yield strength and the indentation yield strength was approximately 1.9. Weaver et al. [7] studied the mechanical response of single-phase α-Ti-64, single-crystal (α-β), few-crystalline and polycrystalline clusters of Ti-64 using spherical indentations, and found that the indentation modulus and the yield strength of multiple indentations remained relatively constant, but decreased with the indenter size. The method of the indentation σ-ε relationship has also been extended to other composites [8,9,10,11,12,13,14,15,16] from the load–displacement data through spherical nanoindentation experiments. In all the studies examined, it can be seen that the appropriate definitions of the contact area, the stress and the strain in the indentation are very important. 

In this paper, based on the former research on spherical indentation, the suitable calculation formulas for the hierarchical honeycomb nanoporous materials in the nanoindentation are selected, uniaxial compression tests on the hierarchical honeycomb nanoporous materials are simulated, and the spherical nanoindentation macroscopic stress–macroscopic strain relationship of the hierarchical materials is derived via the spherical nanoindentation and the uniaxial compression. From the macroscopic stress–macroscopic strain relationships, the macroscopic nanoindentation modulus and the macroscopic nanoindentation yielding strength of the hierarchical nanoporous materials are analyzed using the empirical formulas. These can not only complement existing studies on the hierarchical honeycomb nanoporous materials, but also contribute to further research on this material. 

## 2. Theory

To obtain the stress and the strain relation corresponding to the material from the curve of the nanoindentation force–nanoindentation depth relation, the contact radius a has been calibrated by many researchers [6,17,18,19,20,21] through combining Hertz theory and Sneddon tests to examine the frictionless contact on the two isotropic materials. Further, homogeneous bodies with quadratic surfaces can be calibrated to take into account the complex changes in the sample surface geometry [4] and generally estimated using the spherical nanoindenter as the following equation:

(1) $$a=\sqrt{{2R}_{i}h_{c}-h_{c}^{2}}$$

Here, $R_{i}$ is the nanoindenter radius and $h_{c}$ is the contact penetration calculated by the following equation [22]

(2) $$h_{c}=h_{\max}-\varepsilon \frac{P_{\max}}{S}$$

Here, $h_{\max}$ is the maximal depth, $P_{\max}$ is the maximal force, and ε is 0.75 for the sphere nanoindenter. S is calculated by $S=\frac{dP_{u}}{dh}{|}_{P=P_{\max}}$, and $E_{r}$ is the effective elastic modulus calculated by the following equations:

(3) $$E_{r}=\frac{1}{\beta }\frac{\sqrt{\pi }}{2}\frac{S}{\sqrt{A}}$$

(4) $$\frac{1}{E_{r}}=\frac{1-{\bar{\upsilon }}^{2}}{\bar{E}}+\frac{1-\upsilon_{i}^{2}}{E_{i}}$$

where β is a dimensionless parameter related to the geometry of the nanoindenter (for the circular nanoindenter, β=1). $\bar{E}$ and $E_{i}$ denote the elastic modulus of the specimen and the nanoindenter, respectively. $\bar{\upsilon }$ and $\upsilon_{i}$ are, respectively, the Poisson’s ratio of the specimen and the nanoindenter [22]. 

The contact area A and the nanoindentation stress ${\bar{\sigma }}_{\mathrm{ind}}$ [22,23] are defined as follows:

(5) $$A=\pi a^{2}$$

(6) σ¯ind=PA

Because the distribution of the stress and the strain in the nanoindentation zone is extremely uneven, the nanoindentation representative stress $\bar{\sigma }$ and the nanoindentation representative strain $\bar{\varepsilon }$ of the hierarchical nanoporous material are defined as follows [2,4,5,6,17,18,19,20,21,22,23,24]:

(7) σ¯≈σ¯indC*

(8) ε¯=0.2 aRi

Here, $C^{*}$ is the limiting factor (Patel $C^{*}=2.2$ [18], Weaver $C^{*}=1.9$ [6], Tabor $C^{*}=2.8$ [19], Liu $C^{*}=1.6$ [24] and segmented-function representation [25]). The value of the limiting C* is defined by comparing the results of the nanoindentation and the uniaxial tensile or uniaxial compression tests on the material. C* is decided by the indentation strain and increases with the increasing strain. C* depends on the type of indenters and the materials [18,19,20,24,25].

## 3. Results and Discussion

In this part, the 3D finite-element nanoindentation models of the hierarchical honeycomb nanoporous films are established, uniaxial compression tests on the hierarchical honeycomb nanoporous materials are simulated, and the spherical nanoindentation macroscopic stress–macroscopic strain relationship of the hierarchical honeycomb nanoporous material is defined by the combination of the nanoindentation and the uniaxial compression simulation in the end. Further, the macroscopic elastic modulus $\bar{E}$ and the macroscopic yielding stress $\bar{\sigma_{s}}$ of the hierarchical material are obtained from the stress–strain curve. At the same time, the results are analyzed with the empirical formula.

### 3.1. Nanoindentation on the Hierarchical Honeycomb Nanoporous Materials

**Figure 1:**
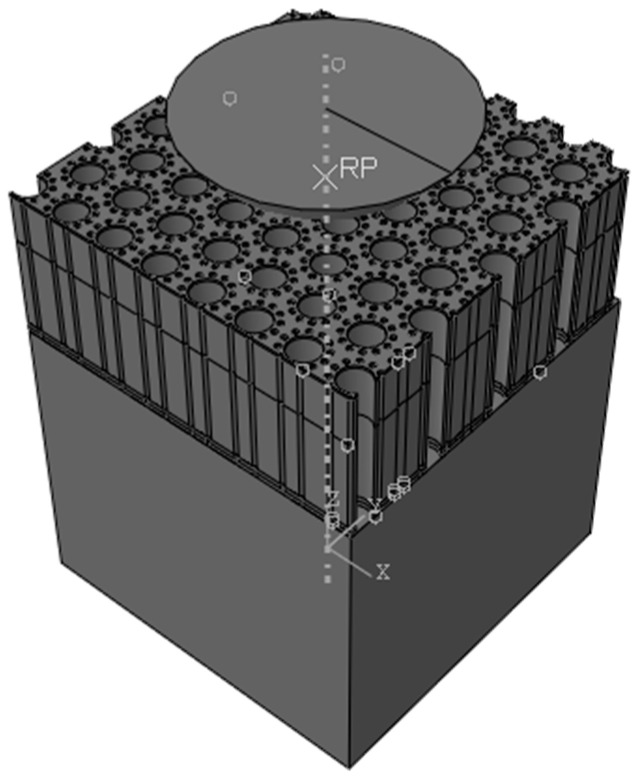
The hierarchical nanoporous material in the nanoindentation.

**Figure 2:**
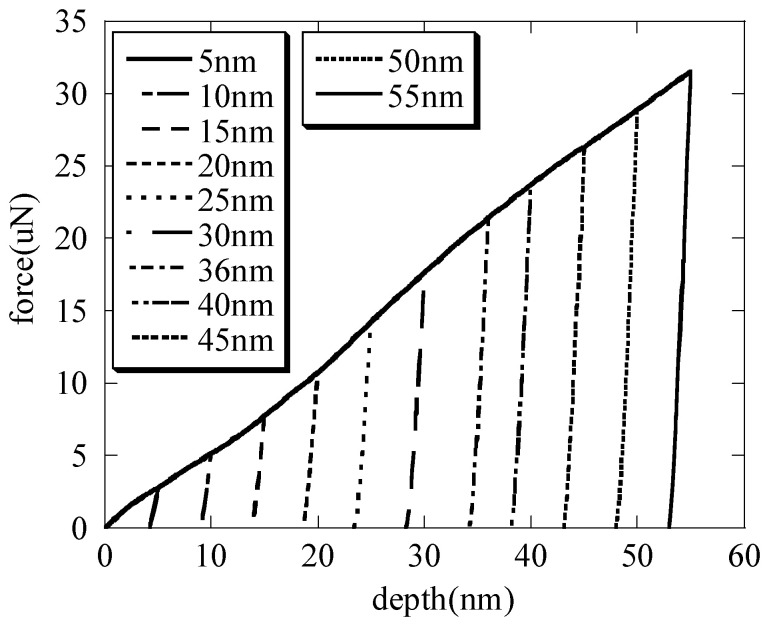
The load-unload curves during the elastic–plastic nanoindentation.

The 3D finite nanoindentation models of the hierarchical honeycomb nanoporous film with a relative density of 54.4% (one big hole R = 49.541 nm, twelve small holes r = 8.777 nm shown in Figure 1) are nanoindented from 5 nm to 55 nm by Abaqus 6.14. The spherical nanoindenter is assumed to be a rigid body ($R_{i}=500\mathrm{nm}$). The hierarchical nanoporous films are simulated using C3D8R elements, and the elements close to the nanoindenter tip are refined to obtain accurate results. The elastic Si substrate (the elastic modulus E = 127 GPa, Poisson’s ratio υ=0.278) is modeled with C3D8R elements. The friction effects between the spherical nanoindenter and the hierarchical nanoporous film are ignored [26]. The film–substrate interface retains perfect integrity. Both the film and the substrate are assumed to be homogeneous and isotropic. The solid material (the elastic modulus E = 55 GPa, Poisson’s ratio $\upsilon_{s}=0.44$, the yielding stress $\sigma_{s}=220\mathrm{MPa}$) of the hierarchical honeycomb nanoporous materials with a film thickness of 500 nm is assumed to be the elastic–perfectly plastic material. In the analysis, the macro Poisson’s ratio $\bar{\upsilon }$ of the hierarchical nanoporous materials is equivalent to Poisson’s ratio $\upsilon_{s}$ of the solid materials [27,28,29,30] and 1−υi2Ei=0 for the rigid nanoindenter. The force–depth curves that are nanoindented with different maximum depths on the specimens are shown in Figure 2.

**Figure 3:**
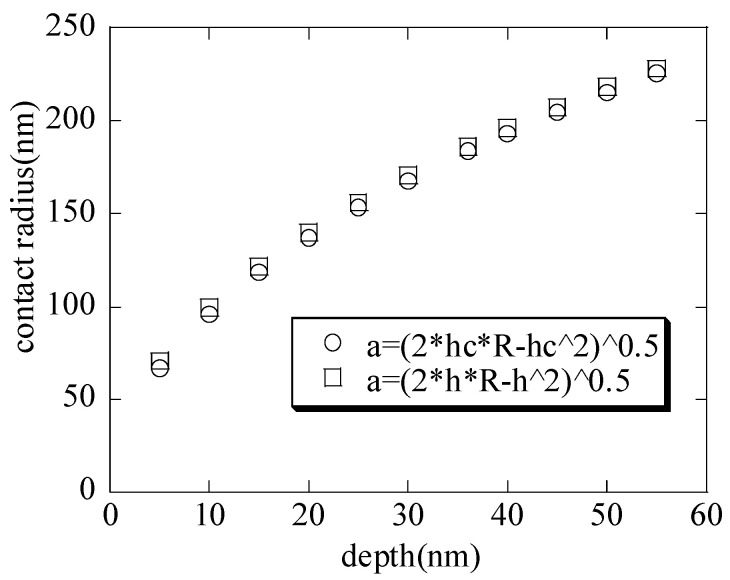
The contact area–maximum depth curves with the increasing depths.

**Figure 4:**
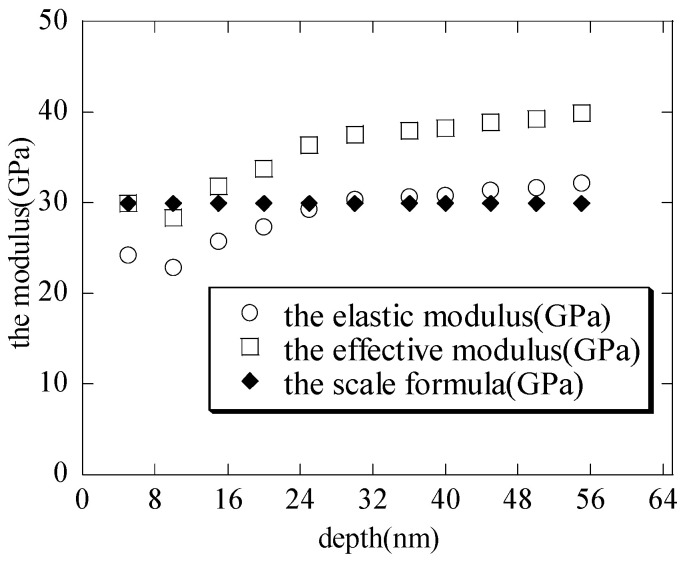
The modulus–maximum depth curves with the increasing depths.

With the increasing maximum depth, the contact radii (shown in Figure 3) are calculated by substituting the contact depth $h_{c}$ and the total depth h into Equation (1), respectively. It is seen that the contact radii are basically the same; thus, the contact radius $a=\sqrt{{2R}_{i}h_{c}-h_{c}^{2}}\approx \sqrt{{2R}_{i}h-h^{2}}$ when the nanoindenter radius is far greater than the depth. This is also consistent with the literature [1]. With the increasing maximum depth, the macroscopic elastic moduli (shown in Figure 4) of the hierarchical nanoporous film are closer to the results of the scale formula ($\bar{E}=E_{s}\rho^{*}$) [27,28,29,30]. It can also be seen that if the nanoindentation depth of the hierarchical nanoporous materials is too shallow, it cannot reflect the macroscopic mechanical properties of the hierarchical nanoporous materials, and if it is too deep, the results of the hierarchical nanoporous materials will be affected by the substrate. Thus, it is necessary to choose the appropriate nanoindentation depth to more accurately reflect the mechanical properties of the hierarchical nanoporous material. From the results in Figure 4, the hierarchical nanoporous film is nanoindented to a depth of 30 nm in the latter analysis.

### 3.2. Uniaxial Compression Simulation Tests on the Hierarchical Honeycomb Nanoporous Materials

**Figure 5:**
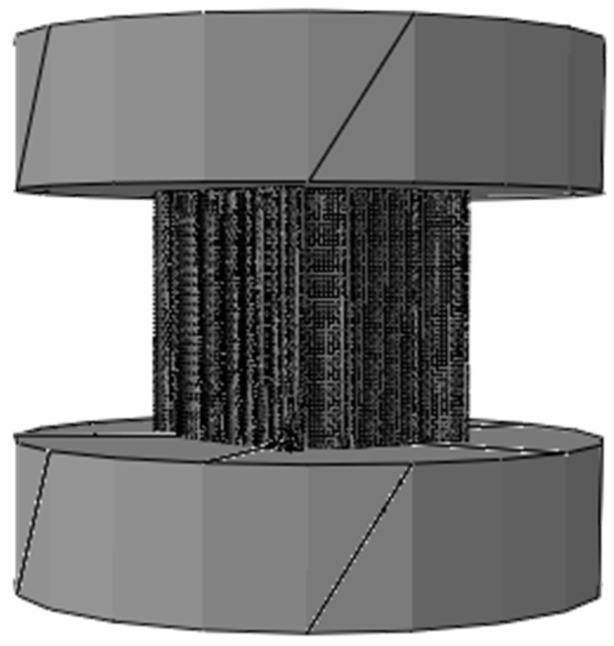
The hierarchical nanoporous material in the uniaxial compression simulation test.

The uniaxial compression tests on the hierarchical honeycomb nanoporous materials (one big hole R = 49.541 nm, twelve small holes r = 8.777 nm shown in Figure 1) with the relative density 54.4% are simulated by Abaqus 6.14 (shown in Figure 5). The solid materials (the elastic modulus E = 55 GPa, Poisson’s ratio $\upsilon_{s}=0.44$ and the different yielding stresses $\sigma_{s}$ from 110 MPa to 620 MPa) of the hierarchical honeycomb specimens are the elastic–perfectly plastic material. In order to ensure the compression on the enough holes and to obtain more accurate macroscopic mechanical properties of the hierarchical nanoporous materials, the size of the specimens is set at 492 nm×436 nm ×500 nm. The hierarchical honeycomb nanoporous specimens are compressed down to 25 nm.

**Figure 6:**
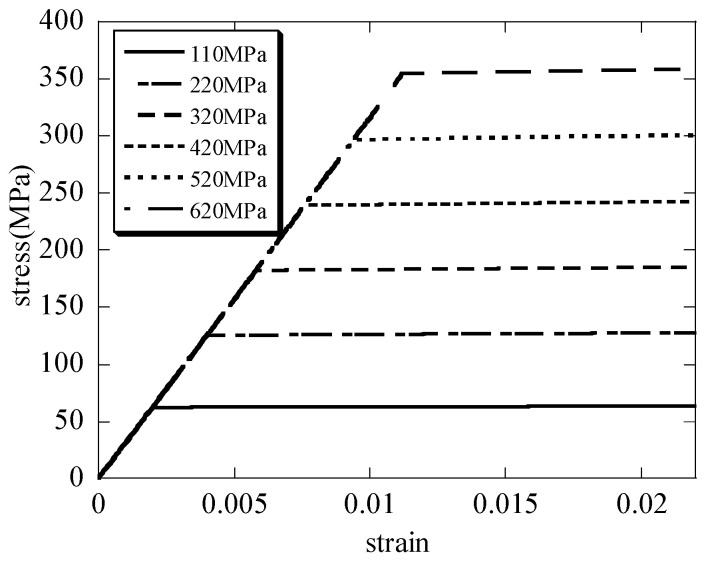
The stress and strain curves during the uniaxial compression simulation tests.

**Figure 7:**
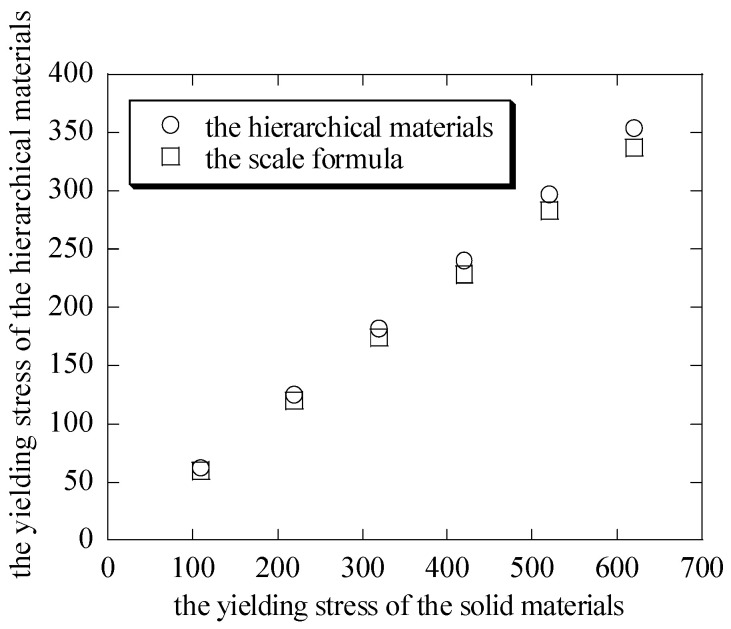
The difference of the yielding stress between the hierarchical materials and the scale formula.

The macroscopic stress and the macroscopic strain curves of the hierarchical honeycomb nanoporous materials are seen in Figure 6. It can be seen that when the solid materials of the hierarchical nanoporous materials are assumed to be elastic–perfectly plastic, the results in Figure 6 have showed the elastic–perfectly plastic behavior when the hierarchical nanoporous materials are uniaxially compressed. From the curves, the differences of the yielding stresses $\bar{\sigma_{s}}$ between the hierarchical materials and the scale formula ($\bar{\sigma_{s}}=\sigma_{s}\rho^{*}$ [27,28,29,30]) are shown in Figure 7. It can be seen that the macroscopic yielding stresses $\bar{\sigma_{s}}$ in the uniaxial compression tests on the hierarchical honeycomb nanoporous materials are close to the results of the scale formula. 

### 3.3. The Determination of the Macroscopic Stress–Macroscopic Strain Relationship on the Hierarchical Honeycomb Nanoporous Materials

**Figure 8:**
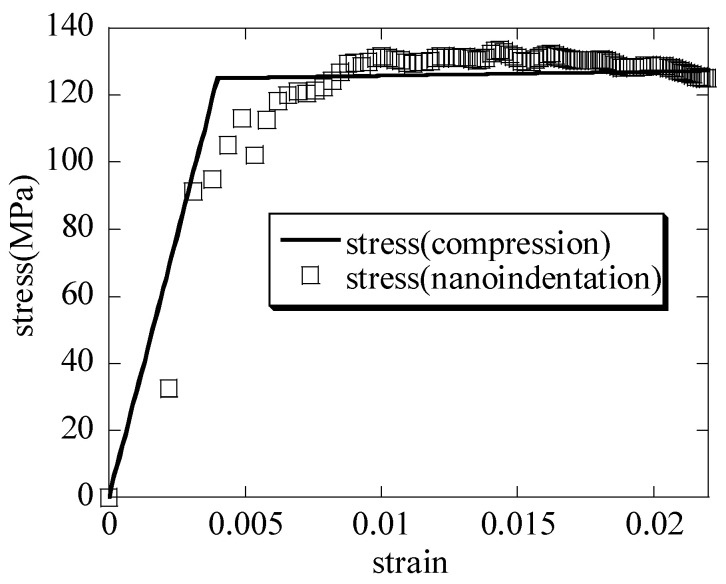
The macroscopic stress–macroscopic strain curves created by combining the nanoindentation and the uniaxial compression.

The hierarchical honeycomb nanoporous material is nanoindented to a depth of 30 nm and is compressed down to 25 nm; the macroscopic stress–macroscopic strain curves of the nanoindentation and the compression are shown in Figure 8. When the nanoindentation-representative stresses of the hierarchical nanoporous material in the nanoindentation are defined, the limiting factor $C^{*}$ shown in Equation (7) is the segmented-function representation given as follows:

$$\left\{\begin{array}{l} C^{*}=1,\varepsilon \leq 0.004\\ C^{*}=1.3,0.004<\varepsilon \leq 0.006\\ C^{*}=1.6,\varepsilon >0.006 \end{array}\right.$$

From the curves, the macroscopic elastic modulus $\bar{E}$ and the macroscopic yielding stress $\bar{\sigma_{s}}$ of the hierarchical nanoporous material by the nanoindentation and the uniaxial compression are obtained, and the results are compared with the scale formula in Table 1. It can be seen that the results are close to one another.

## 4. Conclusions

In this paper, the spherical nanoindentation macroscopic stress–macroscopic strain curve of the hierarchical honeycomb nanoporous material is obtained by combining the spherical nanoindentation and the uniaxial compression. At the same time, the macroscopic elastic modulus and the macroscopic yielding stress of the hierarchical material are obtained from the curves by these methods. The conclusions are derived as follows:

(1) When the nanoindenter radius is far greater than the depth, the elastoplastic contact radius $a(\approx \sqrt{{2R}_{i}h-h^{2}})$ is approximately calculated by the total depth h. This is also consistent with the literature [1]. With the increasing maximum depth, the macro elastic moduli of the hierarchical nanoporous film are close to the results of the scale formula ($\bar{E}=E_{s}\rho^{*}$). 

(2) When the hierarchical materials are uniaxially compressed down to 25 nm, the macroscopic stress and the macroscopic strain curves of the hierarchical honeycomb nanoporous materials which have different yielding stresses $\sigma_{s}$ from 110 MPa to 620 MPa are obtained. The results show that the macroscopic yielding stresses $\bar{\sigma_{s}}$ of the hierarchical honeycomb nanoporous materials are close to the results of the scale formula ($\bar{\sigma_{s}}=\sigma_{s}\rho^{*}$). At the same time, it can be seen that when the solid materials of the hierarchical nanoporous materials are assumed to be elastic–perfectly plastic, the hierarchical nanoporous materials that are uniaxially compressed show the elastic–perfectly plastic behavior.

(3) Taking into account the extremely uneven distribution of the stress and the strain in the nanoindentation zone when the nanoindentation representative stress $\bar{\sigma }$ and the nanoindentation representative strain $\bar{\varepsilon }$ of the hierarchical nanoporous material are defined, the limiting factor $C^{*}$ is represented as the segmented function ($C^{*}=1,\;\varepsilon \leq 0.004;$ $C^{*}=1.3,\;0.004<\varepsilon \leq 0.006;$ $C^{*}=1.6,\;\varepsilon >0.006$). At the same time, the macroscopic stress $\bar{\sigma }$–macroscopic strain $\bar{\varepsilon }$ curve of the hierarchical nanoporous materials nanoindented to a depth of 30 nm is basically consistent with the curve of the hierarchical nanoporous materials that is uniaxially compressed down to 25 nm. Moreover, the macro elastic modulus $\bar{E}$ and the macro yielding stress $\bar{\sigma_{s}}$ of the hierarchical nanoporous material from the two constitutive curves are close to the results of the scale formula ($\bar{E}=E_{s}\rho^{*},\bar{\sigma_{s}}=\sigma_{s}\rho^{*}$).

## Figures and Tables

**Table 1:** The macroscopic elastic modulus and the macroscopic yielding stress of the hierarchical nanoporous material.

Method	$\bar{E}$ (GPa)	$\bar{\sigma_{s}}$ (MPa)
Nanoindentation	26.8	126
Compression	31.3	125
The scale formula [27,28,29,30]	29.9	120

## Data Availability

Data are contained within the article.
